# Stem Cell Selection In Vivo Using Foamy Vectors Cures Canine Pyruvate Kinase Deficiency

**DOI:** 10.1371/journal.pone.0045173

**Published:** 2012-09-13

**Authors:** Grant D. Trobridge, Brian C. Beard, Robert A. Wu, Christina Ironside, Punam Malik, Hans-Peter Kiem

**Affiliations:** 1 Clinical Division, Fred Hutchinson Cancer Research Center, Seattle, Washington, United States of America; 2 Department of Medicine, University of Washington School of Medicine, Seattle, Washington, United States of America; 3 Department of Experimental Hematology, Cincinnati Children’s Hospital Medical Center, Cincinnati, Ohio, United States of America; 4 Department of Pathology, University of Washington School of Medicine, Seattle, Washington, United States of America; University of Frankfurt - University Hospital Frankfurt, Germany

## Abstract

**Background:**

Hematopoietic stem cell (HSC) gene therapy has cured immunodeficiencies including X-linked severe combined immunodeficiency (SCID-X1) and adenine deaminase deficiency (ADA). For these immunodeficiencies corrected cells have a selective advantage in vivo, and low numbers of gene-modified cells are sufficient to provide therapeutic benefit. Strategies to efficiently transduce and/or expand long-term repopulating cells in vivo are needed for treatment of diseases that require higher levels of corrected cells, such as hemoglobinopathies. Here we expanded corrected stem cells in vivo in a canine model of a severe erythroid disease, pyruvate kinase deficiency.

**Methodology/Principal Findings:**

We used a foamy virus (FV) vector expressing the P140K mutant of methylguanine methyltransferase (MGMTP140K) for in vivo expansion of corrected hematopoietic repopulating cells. FV vectors are attractive gene transfer vectors for hematopoietic stem cell gene therapy since they efficiently transduce repopulating cells and may be safer than more commonly used gammaretroviral vectors. Following transplantation with HSCs transduced ex vivo using a tri-cistronic FV vector that expressed EGFP, R-type pyruvate kinase, and MGMTP140K, we were able to increase marking from approximately 3.5% to 33% in myeloid long-term repopulating cells resulting in a functional cure.

**Conclusions/Significance:**

Here we describe in one affected dog a functional cure for a severe erythroid disease using stem cell selection in vivo. In addition to providing a potential cure for patients with pyruvate kinase deficiency, in vivo selection using foamy vectors with MGMTP140K has broad potential for several hematopoietic diseases including hemoglobinopathies.

## Introduction

Pyruvate kinase (PK)-deficiency in the Basenji dog causes severe life-threatening hemolytic anemia [1]. Previous allogeneic transplantation studies have shown that a high (∼20%) percentage of corrected long-term repopulating cells is required to ameliorate the disease phenotype [2], so the PK-deficient dog is an excellent model for hematopoietic diseases that will require a high percentage of corrected cells to achieve therapeutic benefit. Affected dogs suffer from chronic, degenerative, hemolytic anemia with low hematocrits [3], [4]. ^51^Cr-tagged red blood cell (RBC) survival, which in normal dogs averages 1 month, is shortened to a few days [5]. PK-deficient dogs have erythrocyte PK activity mediated by the M2-type PK isoenzyme, which is normally present in all tissues during fetal life and remains the major isoenzyme in erythroid precursors [6]. These dogs lack the normal R-type, which begins to appear in normal erythrocytes as erythroid maturation proceeds [1]. The expression of the M2-type isoenzyme is thought to compensate for R-type PK deficiency but it does not prevent hemolysis in vivo. PK-deficiency in humans is highly variable and symptoms range from a severe hemolytic anemia requiring transfusions, to a fully compensated hemolytic process without anemia. In severe cases the disease can be fatal in early childhood. We undertook studies in the dog model not only for gene therapy for PK-deficiency, but also as a model for more prevalent erythroid diseases such as β-thalassemia, which requires a high percentage of corrected cells to achieve a therapeutic benefit [7], [8].

Tani, et al. [9] introduced the human PK enzyme into mouse bone marrow cells using a gammaretroviral vector that expressed human PK from an SV40 promoter. Although these authors demonstrated expression of human PK mRNA by PCR in mouse peripheral blood following transplantation, they did not observe long-term expression. This is likely because of inefficient transduction of the hematopoietic cells in this early study, and also low expression of PK in hematopoietic cells from the SV40 promoter. A gene therapy approach was also developed where normal PK-expressing murine cells were transduced with a vector that allowed in vivo expansion using a chemical inducer of dimerization after transplantation into PK-deficient mice [10]. In this study correction of anemia was observed with a donor chimerism of 10%, indicating that anemia can be corrected when 10% of engrafted cells express PK from the endogenous mouse PK gene. In vivo expansion of gene modified cells also improved anemia, but the effects were transient, with in vivo expansion occurring in erythroid cells, not in HSCs due to the design of the dimerization construct. More recently the human R-type PK gene was used to correct PK deficiency in mice [11] in a gene therapy approach. In this study lineage-negative mouse bone marrow (BM) cells were transduced with a gammaretroviral vector expressing human R-type PK resulting in resolution of the hematological symptoms of the mouse model. In this model long-term correction of the disease phenotype was dependent on the percentage of corrected repopulating cells, requiring approximately 25% of cells. An in utero gene therapy approach was less efficient in this study resulting in only partial correction.

Our approach was to use a foamy virus (FV) vector with a P140K mutant methylguanine DNA methyltransferase resistance gene (MGMTP140K) in addition to the therapeutic canine PK-R transgene. FV vectors can transduce pluripotent murine [12] and also human [13]–[15] hematopoietic repopulating cells as assessed in murine models where human cells are transduced ex vivo and transplanted into immunodeficient mice [16]. FV vectors [13], [17]can also efficiently transduce canine long-term repopulating cells [18], [19]. They have a unique integration profile, integrating near promoters less frequently than MLV vectors, and within genes less frequently than HIV-derived vectors [20]. An unfortunate property of retroviral vectors in regards to their use for gene therapy is that integrated vector proviruses can dysregulate nearby proto-oncogenes. This has led to vector-mediated T cell leukemia in SCID-X1 gene therapy trials, and also myeloid dysplasias in a chronic granulomatous disease clinical trial. In a direct comparison with gammaretrovirus and lentiviral vectors, FV proviruses also had a reduced propensity to transactivate nearby genes [21]. Together, these properties suggest that FV vectors may be relatively safe, and are an attractive alternative to the gammaretroviruses used in the above clinical trials. MGMTP140K confers resistance to methylating agents such as temozolomide as well as to nitrosoureas such as BCNU, and mediates efficient selection of long-term repopulating HSCs [22], [23].

The MGMT protein is expressed in normal human tissues, so in order to enhance selection a potent inhibitor, O^6^-benzylguanine (O6BG), is used to inactivate endogenous MGMT. The mutant MGMTP140K is not inactivated by O6BG. MGMTP140K-mediated selection in the canine HSC gene therapy model provides durable selection at the stem cell level leading to amplification in multiple hematopoietic lineages [24]. Our goal was to efficiently transduce long-term repopulating HSCs and then select them in vivo after transplantation to increase the percentage of corrected cells to cure canine PK-deficiency [25].

## Results

### Development of FV PK Vector with MGMTP140K

**Figure 1:**
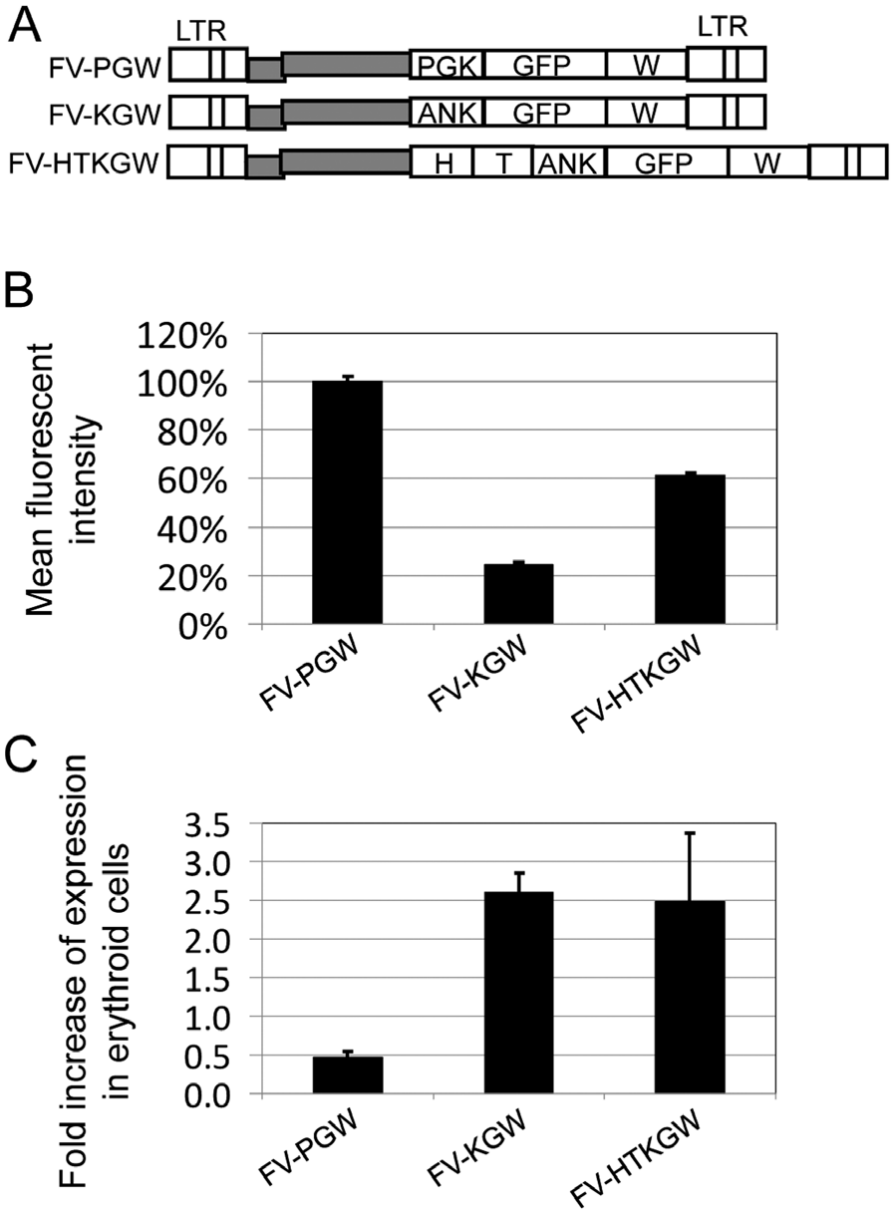
FV erythroid vectors. a) Foamy EGFP vectors containing the phosphoglycerate kinase promoter FV-PGW, minimal ankyrin promoter FV-KGW, and the enhanced ankyrin with the HS-40 (H) and GATA-1 (T) promoter-enhancer elements FV-HTKGW. W is the woodchuck hepatitis virus post-transcriptional regulatory element. LTR is the long terminal repeat. **b)** The relative expression level of EGFP in hemin-induced K562 cells which undergo erythroid differentiation is shown as determined by mean fluorescent intensity of EGFP expression by flow cytometry relative to the FV vector with the PGK promoter, FV-PGW. **c)** The ability to specifically express a transgene in erythroid cells was evaluated by comparing EGFP expression in hemin-induced K562 cells and non-erythroid HT1080 fibrosarcoma cells is shown. The percentage of cells expressing transgene is shown. As expected, the ankyrin promoters exhibited erythroid-specific expression. **d)** Flow cytometry analysis was performed to confirm erythroid-induction of K562 cells by evaluating expression of the erythroid markers CD71 (transferrin) and CD235a (glycophorin) on K562 cells after 7 days of culture with 50 mM hemin and 3×10^−8^ M aclacinomycin (blue) or untreated (red). Increased expression of CD71 and CD235a were observed as a result of erythroid induction. NS = not stained; NTC = non-transduced control. **e)** Hemin induction of hemoglobin expression in K562 cells was confirmed by benzidine staining. Cells were cultured untreated for 7 days (left panel) or with 50 mM hemin and 3×10^−8^ aclacinomycin (right panel). Efficient induction of hemoglobin expression was observed as indicated by blue benzidine staining in induced cells.

We previously demonstrated that a FV vector with an internal housekeeping phosphoglycerate kinase (PGK) promoter expresses enhanced green fluorescent protein (EGFP) in canine RBCs and platelets in vivo [18]. This suggested that the PGK promoter might be effective for gene therapy of RBC diseases. Previous studies in mice suggested the human ankyrin promoter may also be effective for erythroid expression [26], [27]. We developed erythroid FV vectors with minimal and enhanced human ankyrin promoters and compared transgene expression in erythroid cells to the PGK promoter (**Figure 1**). As expected the ankyrin promoters provided erythroid-specific expression, however the housekeeping PGK promoter provided the highest level of erythroid transgene expression, and was chosen for the in vivo studies.

**Figure 2:**
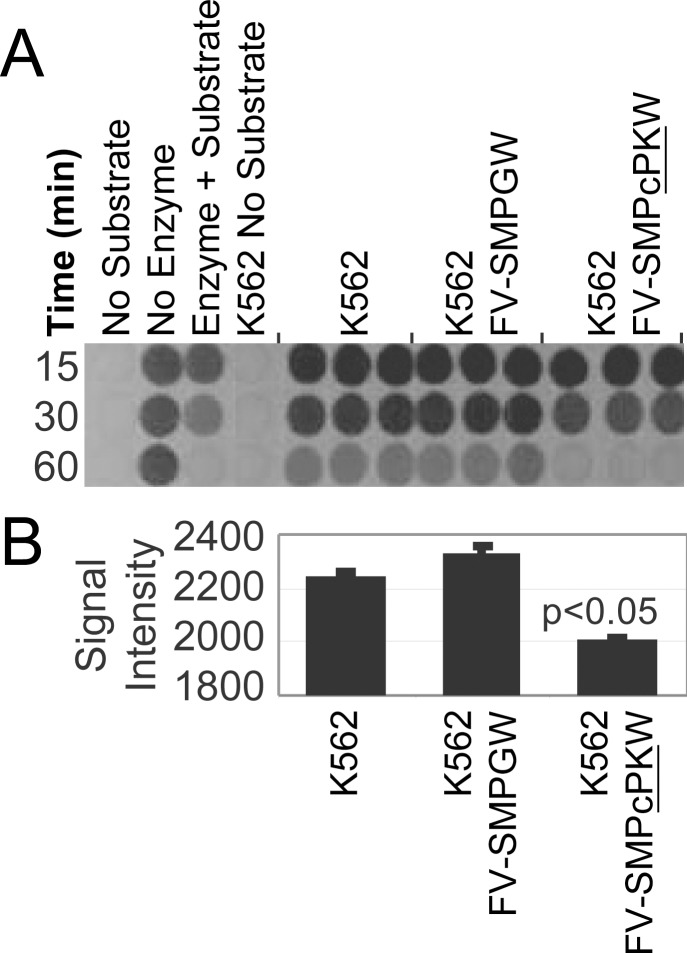
PK expression from FV vectors. **a)** Fluorometric assay for pyruvate kinase expression from the foamy canine PK vector FV-SMPcPKW. The presence of pyruvate kinase enzyme is detected by the conversion of fluorescent NADH to non-fluorescent NAD^+^. In this assay adenosine diphosphate (ADP) and phosphoenolpyruvate are converted to adenosine triphosphate (ATP) and pyruvate by pyruvate kinase (PK). Pyruvate and fluorescent NADH are then converted to lactate and non-fluorescent NAD+ by lactate deydrogenase as indicated by a reduction in fluorescence over time from 15 minutes to 60 minutes. “No Substrate” is no PK assay components, “No Enzyme” is all assay components but no positive control of purified PK enzyme. “Enzyme + Substrate” is all assay components with purified PK enzyme as a positive control with no cell lysate. “K562 No Substrate” is K562 cell lysate with lactate dehydrogenase but no phosphoenolpyruvate, ADP or NADH. “K562” is K562 cell lysate with all assay components in triplicate. “K562 FV-SMPGW” is cell lysate from K562 cells transduced with the negative control vector FV-SMPGW that does not express PK with all assay components in triplicate. “K562 FV-SMPcPKW” is cell lysate from K562 cells transduced with the vector FV-SMPcPKW that expresses canine PK with all assay components in triplicate. In K562 cells transduced with the FV vector expressing canine PK there is a more rapid reduction in fluorescence compared to K562 cells or K562 cells transduced with a control FV vector that does not express PK. **b)** Densitometry analysis of loss of fluorescence at 30 minutes shows the FV-SMPcPKW vector that expresses canine PK significantly reduces the amount of fluorescent PK substrate relative to the control vector FV-SMPGW that does not express PK (p<0.05).

The canine PK R-type transgene was synthesized based on available published sequences and cloned into a previously described FV vector, FV-SMPGW [17]. The resulting bi-cistronic FV-SMPcPKW vector expresses MGMTP140K driven by a spleen focus forming virus (SFFV) promoter and canine PK R-type expressed from a PGK promoter. K562 erythroid cells were transduced with the canine PK vector or the control FV-SMPGW vector, and then treated with O6BG and BCNU to increase the percentage of transduced cells. Following selection, the level of PK activity was determined by the method of Beutler, et al. [28]. (**Figure 2**). The FV-SMPcPKW vector mediated over-expression of PK (p<0.5**)**, confirming the function of the PK-R transgene. However, the absence of a reporter gene made it difficult to accurately evaluate the efficiency of MGMTP140K-mediated selection. We thus modified the FV-SMPcPKW vector to also express EGFP by inserting a foot and mouth disease virus (FMDV) 2A sequence 3' to the MGMTP140K transgene to create the tri-cistronic vector FV-SM2AGPcPKW (**Figure S1a**) which allowed us to conveniently track selection of corrected cells using EGFP (**Figure S1b**). The transduction efficiency of this vector as assessed by transgene expression (EGFP) and by real-time PCR was similar to the control vector FV-SMPGW and efficient in canine CD34^+^ cells (**Figure S1c).**

### FV Gene Therapy in the Basenji Dog Model

Our approach was to transplant a PK-deficient dog with autologous CD34^+^ hematopoietic cells transduced with the FV-SM2AGPcPKW vector, select for corrected cells using the MGMTP140K transgene, and monitor selection using EGFP. One of the characteristics of the canine PK disease model is anemia with a high nucleated RBC count, presumably due to the high turnover of PK-deficient RBCs. In PK dogs nucleated RBC counts are elevated to compensate for the loss of RBCs by hemolysis. Thus, phenotypic correction can be easily and routinely monitored by complete peripheral blood (PB) counts that report both nucleated and mature RBCs.

**Figure 3:**
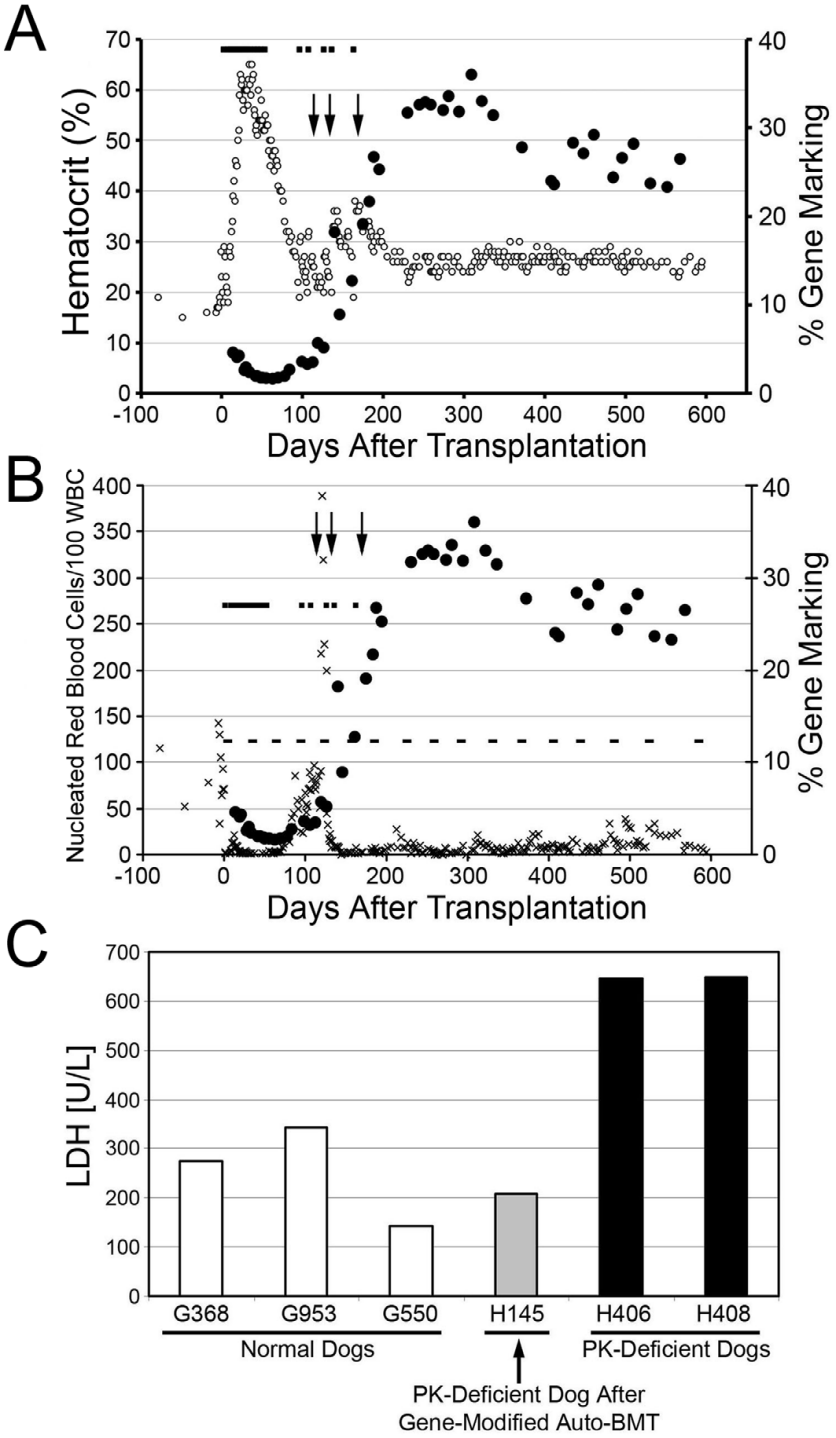
Gene marking, *in vivo* selection, and correction of pyruvate kinase (PK)-deficiency indicated by stabilizing hematocrit, reduction in nucleated red blood cells (RBCs) and lactate dehydrogenase (LDH) following transplantation of a PK-deficient dog with FV vector-modified cells. a ) The percentage of EGFP expressing granulocytes (•) determined by flow cytometry, hematocrit (○), and transfusions (▪). Treatment with O6BG and BCNU (↓) leading to stable increases in the percentage of gene modified cells. **b**) The percentage of EGFP expressing granulocytes (•) determined by flow cytometry, nucleated RBCs (x), dashed line is an extrapolation of the average nucleated RBCs per 100 white blood cells (WBCs) of PK-deficient dogs prior to transplantation, and transfusions (▪). Following transplantation transfusions were initially necessary to maintain a hematocrit of 20% (**a**) and reduce nucleated RBCs (**b**). After the third treatment with O6BG and BCNU the hematocrit has been maintained over 20%. Nucleated RBCs have remained low for over 1 year indicating phenotypic correction of PK deficiency. (**c**) Reduction of lactate dehydrogenase (LDH) levels, indicating reduced RBC lysis after gene therapy.

A single basenji dog, H145, was identified that had a low hematocrit, typically below 25 and frequently below 20, with high reticulocyte counts. H145 was confirmed as PK^−/−^ by PCR. PB CD34^+^ cells were harvested from H145 and transduced with the foamy vector at a multiplicity of infection of 9.9 (**Table 1**). A myeloablative preparative regimen was used to avoid an immune response to the EGFP protein, human MGMTP140K, and canine PK-R gene, which are all foreign to PK^−/−^ dogs. Following infusion of vector-exposed cells, marking was evaluated in PB granulocytes (**Figure 3a** **)**. The marking stabilized at approximately 3.5% EGFP-expressing granulocytes by 100 days after transplantation. At this time 0.4% of peripheral blood lymphocytes expressed EGFP (data not shown). The hematocrit was elevated due to whole blood transfusions required for platelet support following transplantation, but following cessation of transfusions for platelet support it was determined that the dog was not able to maintain hematocrit in a normal range without transfusions. Because of this we administered three treatments of O6BG and BCNU on days 114, 133, and 169 after transplantation to select for corrected, MGMTP140K-expressing cells. Marking in both myeloid and lymphoid lineages increased after the treatments as evidenced by an increase in EGFP-expressing PB granulocytes and lymphocytes (**Figure 3a**) and marking was observed in all lineages examined (**Figure 4**). After the third treatment, the percentage of EGFP-expressing cells stabilized at approximately 33% in granulocytes and 5.5% in lymphocytes. Prior to, and during the first two treatments, the dog required whole blood cell transfusions to treat low platelets and anemia as indicated by a drop in hematocrit to below 20% (**Figure 3b**). However, after the third treatment the hematocrit declined slightly but did not drop below 22% and stabilized at approximately 25% with an associated decrease in nucleated RBC, typically between 2 and 4 nucleated RBC/100 white blood cells, confirming therapeutic benefit (**Figure 3b**). Thus, after increasing the percentage of gene-modified cells, this dog has become transfusion-independent. Serum levels of lactate dehydrogenase (LDH) were tested to monitor RBC lysis and the PK-affected dog transplanted with the gene-modified cells showed levels similar to normal dogs and lower levels than PK-affected dogs (**Figure 3c**).

**Table 1:** Transduction and engraftment of canine CD34^+^ cells.

Dog	No. of CD34-enriched cells/kg×10^6^before culture	Purity of CD34-enrichedcells	Number ofcells/ml	Amount of virus/mL†	MOI$	No. of infusedcells/kg×10^6^	Days to ANC >500	Days to platelets >50,000
H145	7.2	57%	5×10^5^	4.6×10^6^	9.9	2.9	9	68

† The amount of virus is the final concentration used during vector exposure based on a functional assay for EGFP transducing units assessed on HT1080 fibrosarcoma cells.

$ Cells were exposed to FV vector at an MOI of 9.9 transducing units/cell for 2 hours and then additional FV was added at an MOI of 9.9 for 20 more hours.

Abbreviations: ANC, absolute neutrophil count; CFU, colony-forming unit; MOI, multiplicity of infection.

**Figure:**
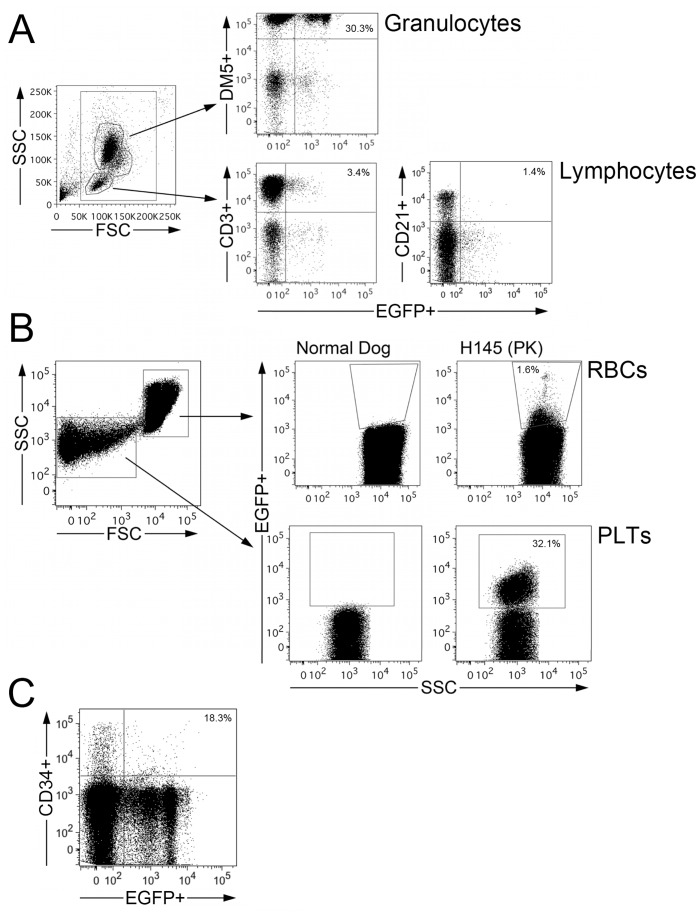
Flow-cytometric analysis of transgene expression in cell subpopulations. In **a)** the percentage of EGFP-expressing cells in granulocyte (DM5) and lymphocyte (CD3 and CD21) subpopulations is shown for dog H145. EGFP-expressing cells were found in all lineages examined. **b)** Gating on red blood cells (RBCs) and platelets (PLTs) was based on scatter characteristics (SSC is side scatter height and FSC is forward scatter height**)** and EGFP-expression is plotted for a control animal and for H145. **c)** The percentage of EGFP-expressing CD34+ cells from the bone marrow is shown for dog H145. EGFP-expressing cells were found in all lineages examined.

The dog is energetic and although it still has a lower than normal hematocrit, it no longer requires transfusions. Thus, following a multi-lineage MGMTP140K-mediated increase in corrected EGFP-expressing cells, we observed therapeutic benefit as evidenced by a stable hematocrit, a stable reduction in nucleated RBCs, lowered lactate dehydrogenase (LDH) levels indicating reduced RBC lysis, and transfusion independence. The treatments with the endogenous MGMT inhibitor, O6BG, and the alkylating agent, BCNU, were well tolerated with only transient transaminitis that resolved quickly, even in this setting of a severe erythroid disease in a large animal model.

## Discussion

Gene therapy for several monogenic red blood cell disorders including hemoglobinopathies will require relatively high percentages of gene-modified cells for therapeutic benefit. Here, we have shown for the first time in a one affected dog that HSC gene therapy with MGMTP140K-mediated in vivo selection can provide a functional cure for a severe erythroid disease that requires relatively high levels of corrected cells in a clinically relevant large animal model. In this model we have combined the MGMTP140K transgene with the therapeutic PK transgene and demonstrated efficient and safe in vivo selection to increase gene marking. Following a multi-lineage increase in gene marking mediated by MGMT-P140K, we observed therapeutic benefit as evidenced by a stable hematocrit, a stable reduction in nucleated red blood cells and transfusion independence.

We observed stable and high marking after three treatments with O6BG and BCNU in both lymphoid and myeloid repopulating cells. Marking in erythroid cells was lower, but we previously observed a lower percentage of EGFP-expressing mature red blood cells relative to granulocytes in both the dog and monkey models, presumably due to the fact that red blood cells are enucleated and the expression of EGFP is largely dependent on its half-life [18], [29]. Thus, marking in erythroid progenitors we report here is also likely underestimated. Immune responses against the transgene(s) are a concern in gene therapy. Both humoral and cytotoxic lymphocyte (CTL) responses to gene-modified cells have been reported after transplantation of EGFP-expressing CD34^+^ cells following a non-myeloablative conditioning regimen or against EGFP/EYFP in baboons after a fully myeloablative conditioning regimen [30]. In this study, cyclosporine was therefore included as an immunosuppressive drug after transplantation to prevent potential immune responses to the gene-modified cells.

In conclusion, we report efficient transduction and selection of long-term, multipotent canine repopulating cells in a PK-deficient dog using FV vectors resulting in phenotypic correction of PK deficiency. The selection was well tolerated and suggests that for severe erythroid diseases that require high levels of gene marking MGMTP140K may be an effective therapeutic approach. Clearly, long-term data is needed to determine the potential toxicity of the alkylating agent BCNU, and the dog model we describe here should provide excellent preclinical data in that regard. Foamy virus integration site analysis is important to determine the safety of this approach and the effects of in vivo selection on the clonal repertoire. These studies are ongoing and will be reported elsewhere. In future studies we plan to test the efficacy of vectors without EGFP that express only MGMTP140K and a therapeutic transgene and could be used in clinical trials. The in vivo selection approach we used may be of therapeutic benefit for patients with PK deficiency. Splenectomy can benefit some PK deficiency patients with severe disease, but allogeneic transplantation is required in some cases [31]. A gene therapy approach using bi-cistronic FV PK and MGMTP140K vectors may be an effective and safe treatment for PK patients who do not have a suitable HLA-matched donor. Additionally, FV HSC gene therapy using in vivo selection with MGMTP140K may be effective for a wide range of severe monogenic erythroid diseases including hemoglobinopathies.

## Materials and Methods

### Ethics Statement

This study was carried out in strict accordance with the recommendations in the Guide for the Care and Use of Laboratory Animals of the National Institutes of Health. The protocol was approved by the Institutional Animal Care and Use Committee of the Fred Hutchinson Cancer Research Center under protocol 1289. All efforts were made to ameliorate suffering.

### Cell Cultures

Human K562 erythroleukemia cells [32] were maintained in RPMI medium 1640. Human HT-1080 [33] cells and human embryonic kidney 293 cells were cultured in Dulbecco’s modified Eagle’s medium (DMEM). All growth media were supplemented with 10% heat-inactivated (56°C for 30 minutes) fetal bovine serum (FBS; HyClone, Logan, UT), 1.25 µg/ml amphotericin, 100 U/ml of penicillin, and 100 µg/ml of streptomycin. Cultures were grown at 37°C in a 5% CO_2_ atmosphere.

### FV Vectors

The FV vector plasmid pFV-SMPGW has been previously described [17] and contains an EGFP reporter transgene expressed from a human phosphoglycerate kinase (PGK) promoter and the MGMTP140K transgene expressed from a SFFV promoter. The pFV-PGW vector plasmid was derived from the pFV-SMPGW plasmid by removing the SFFV-MGMT expression cassette by standard cloning techniques. A human minimal ankyrin promoter was PCR-amplified from HEK 293 cell DNA using primers 5′-GCATGCGACCCCGGGGCAACCAGGGGTC-3′ and 5′-ACCGGTCAGCAGGGGCCCGCCGAAGGGC-3′ and Phusion high-fidelity polymerase (New England Biolabs, Ipswitch, MA), according to the manufacturer’s protocol, and inserted into the FV-PGW vector plasmid replacing the PGK promoter to create the pFV-KGW vector plasmid. The sequence of the PCR-amplified minimal ankyrin promoter was confirmed by sequencing. The enhanced ankyrin vector FV-HTKGW were constructed by replacing the PGK promoter of FV-PGW with an enhanced ankyrin promoter containing the HS-40 erythroid promoter and GATA-1 promoter from previously described lentiviral vector HTKGW [34] using standard cloning techniques. The canine PK sequence was synthesized by Blue Heron Biotechnology (Bothell, WA) using GeneMaker technology according to the sequence from the published canine genome, as obtained from the canine genome browser [35], [36], and inserted into the pFV-SMPGW plasmid replacing the EGFP transgene using standard cloning techniques to create the bi-cistronic vector plasmid pFV-SMPcPKW that expresses the canine PK from the PGK promoter. The tri-cistronic vector plasmid pFV-SM2AGPcPK was constructed by inserting a foot-and-mouth disease 2A sequence and EGFP transgene 3' the MGMT transgene. Vector preparations were generated using PEI-mediated transfection as previously described [17], concentrated 100-fold by centrifugation, and then 5% DMSO was added prior to freezing as a cryoprotectant. These frozen vector preparations were thawed, and the DMSO was removed by dialysis with cell culture media using a Microcon Ultracel YM-50 Centrifugal Filter (Millipore, Billerica, MA) just prior to use. Vector preparations were titered by determining the number of EGFP transducing units on human HT1080 fibrosarcoma cells.

### Analysis of Erythroid-specific Expression

Equal volumes of FV vector preparations were added to K562 cells and HT1080 cells and cultured at 37°C in a 5% CO2 atmosphere. EGFP transgene expression was analyzed by flow cytometry 10 days after vector exposure. Erythroid differentiation of K562 cells was induced 7 days after vector exposure by plating cells at 2×10^5^ cells/mL and culturing in media supplemented with 50 µM hemin (Sigma) and 3×10^−8^ M aclacinomycin (Enzo Life Sciences,Plymouth Meeting, PA) for 7 days. On day 7 of induction, hemoglobin expression was assessed by pelleting and resuspending cells in 0.2% benzidine dihydrochloride (Sigma) in 3% glacial acetic acid and 0.7% hydrogen peroxide, and visualizing using a confocal microscope. EGFP expression and induction was also assessed by staining with antibodies against CD36, CD71 and CD235a conjugated to RPE and analyzing by flow cytometry.

### Analysis of PK Activity and MGMT-mediated Selection

FV-transduced K562 cells were evaluated for PK activity using the method of Beutler [28] that indirectly detects pyruvate kinase activity by measuring conversion of fluorescent NADH to non-fluorescent NAD^+^. Briefly, K562 cells were lysed using Passive Lysis Buffer (Promega) and incubated with 4.5 mM phosphoenolpyruvate (MP Biomedicals), 3 mM adenosine diphosphate (Sigma), and 1.5 mM of reduced nicotinamide adenine dinucleotide (NADH, Sigma) in 2.5 mM potassium phosphate buffer, pH 7.4. At the indicated time points, the reaction was stopped by adding the reaction to Whatman paper, and loss of fluorescence was detected using 365 nm ultraviolet light. MGMT-mediated selection in HT1080 fibrosarcoma cells was performed using final concentrations of 50 µM O6BG and 50 µM BCNU.

### Analysis of Gene Expression and Provirus Copy Number in Colony-forming Units (CFUs)

CD34-enriched cells were cultured in a double layer agar culture system in alpha minimal essential medium supplemented with FBS (Hyclone, Logan, UT), bovine serum albumin (BSA; fraction V, Sigma, St. Louis, MO), 0.5% (wt/vol) agar (Difco, Detroit, MI), overlaid on medium with 0.3% agar (wt/vol) containing 100 ng/mL of cSCF, cG-CSF, canine granulocyte-macrophage colony-stimulating factor (cGM-CSF) and 4 U/mL erythropoietin. Cultures were incubated at 37°C in 5% CO_2_ and 95% air in a humidified incubator. The total number as well as the number of EGFP-positive colonies were enumerated at day 14 of culture by fluorescence microscopy. Provirus copy numbers were determined by measuring EGFP gene levels with the TaqMan 5' nuclease quantitative real-time PCR assay. 300 ng of genomic peripheral blood leukocyte DNA was amplified at least in duplicate with a EGFP-specific primer/probe combination (5′-CTG CAC CAC CGG CAA-3′ and 5′–GTA GCG GCT GAA GCA CTG-3′, probe: 5'-FAM-CCA CCC TGA CCT ACG GCG TG -TAMRA-3′; Synthegen, Houston, TX). A canine IL-3 specific primer/probe combination (5′– ATG AGC AGC TTC CCC ATC C -3′, 5′– GTC GAA AAA GGC CTC CCC -3′, probe 5′–FAM-TCC TGC TTG GAT GCC AAG TCC CAC -TAMRA-3′) was used to adjust for equal loading of genomic DNA.

### Canine Transplantation

The dog H145 was raised and housed at the Fred Hutchinson Cancer Research Center (FHCRC) under conditions approved by the American Association for Accreditation of Laboratory Animal Care. The dog was provided with commercial chow and chlorinated tap water ad libitum and the stem cell transplant was performed when the dog was 384 days old. In preparation for the harvest of stem/progenitor cells, the dog received recombinant canine granulocyte-colony stimulating factor (cG-CSF, 5 µg/kg body weight subcutaneously, twice daily) and recombinant canine stem cell factor (cSCF, 25 µg/kg body weight subcutaneously, once daily) for 5 consecutive days. Leukapheresis was performed using the COBE BCT Spectra Apheresis System via a dual-lumen venous catheter. The machine was primed with cross-matched irradiated donor blood. During the procedure, the dog was constantly monitored for level of sedation or signs of distress, and a slow infusion of 10% calcium gluconate was given to prevent cramping.

In preparation for transplantation, the dog received a single myeloablative dose of 920 cGy total body irradiation (TBI) administered from a linear accelerator at 7 cGy/minute. The animal received broad-spectrum antibiotics and recombinant cG-CSF after transplant until an absolute neutrophil count (ANC) was >1000 µL for two consecutive days. The dog also received cyclosporine to inhibit immune responses to the EGFP transgene from the day before transplant to 35 days after the transplant.

The CD34 enrichment method has been described previously [37], [38]. Briefly, cells were labeled with biotinylated monoclonal antibody 1H6 (IgG1 anti-canine CD34) at 4°C for 30 minutes. The cells were washed twice and then incubated with streptavidin-conjugated microbeads for 30 minutes at 4°C, washed, and then separated using an immunomagnetic column technique (Miltenyi Biotec, Auburn, CA) according to the manufacturer’s instructions.

PB CD34-enriched cells were prestimulated overnight in Iscove’s modified Dulbecco’s medium (IMDM) supplemented with 10% FBS (GIBCO BRL), 1% sodium pyruvate, 1% L-glutamine, 1% penicillin/streptomycin (GIBCO BRL) in the presence of cG-CSF, cSCF, fms-like tyrosine kinase 3 ligand (Flt3-L), and thrombopoietin (TPO) at a concentration of 50 ng/mL each. The following day cells were replated on non-TC treated 75 cm^2^ canted-neck flasks (Corning, Corning, NY) coated with CH-296 (RetroNectin™, Takara Shuzo, Otsu, Japan) at a concentration of 2 µg/cm^2^ with the above media and growth factors. Cells were exposed to FV at an MOI of 9.9 transducing units/cell for 2 hours and then additional FV was added at an MOI of 9.9 for 20 more hours. After transduction, nonadherent and adherent cells were pooled, counted and infused intravenously into the animal.

### In vivo Analysis

EGFP-expressing white blood cells were quantitated by flow cytometric analysis of at least 250,000 events (propidium iodide [1 µg/mL]-excluding, forward and right-angle light scatter-gated) on a FACS Vantage (Becton Dickinson, San Jose, CA). For analysis of RBCs and platelets, a FACSCalibur flow cytometer was used (Becton Dickinson). Flow cytometric data were analyzed by CELLQuest v3.1f software with gating to exclude fewer than 0.1% control cells in the relevant region. The results were then plotted over time in an MS Excel chart. Murine anti-human monoclonal antibodies conjugated to phycoerythrin (PE) and shown to bind to canine CD antigens were used to detect CD21 (clone CA2.1D6, SeroTec) for B cells, and CD14 (clone TÜK4, DAKO) for monocytes. The monoclonal antibody DM5 used to detect granulocytes and the anti-CD3 (clone 17.6B3) used for T cells were kindly provided by Drs. Peter Moore and Brenda Sandmaier. Blood serum levels of lactate dehydrogenase (LDH) were monitored by Phoenix Central Laboratory, Everett, WA. Nucleated red blood cells counts were determined using manual differential of whole blood smears.

### MGMT-mediated in vivo Selection Following Transplantation

Drug treatments were initiated after the dog recovered from transplantation and had stable platelets, >100,000/µl, without transfusion support. Fifty milligrams of O6BG (Sigma Aldrich) were dissolved in 30 ml of 40% polyethylene glycol in PBS, and the concentration was adjusted to 1 mg/kg with pre-warmed (37°C) PBS. This mixture was sonicated for 40 minutes (Branson 3510 sonicator). The drug was further diluted in normal saline to a final volume of approximately 150–200 mL and was infused over 15–20 minutes. The dose of O6BG for the dog was 5 mg/kg. BCNU (Sigma Aldrich) was prepared as previously described [25] and administered 45–60 minutes after the end of the O6BG infusion. Due to potentially underlying liver abnormalities given the genetic background of this dog, we started with a conservative dose of BCNU, 0.2 mg/kg, before escalating to 0.3 mg/kg and then 0.4 mg/kg. Whole blood cell counts and blood chemistry values (including liver and kidney functions) were monitored the day of drug treatment and twice a week thereafter until values returned to pre-treatment levels.

## Supporting Information

Figure S1 MGMT-mediated selection in vitro with a tri-cistronic FV vector. **a)** The FV-SMPGW and tri-cistronic FV-SM2AGPcPKW vectors contain the spleen focus-forming virus (SFFV) promoter driving MGMTP140K expression and the phosphoglycerate kinase (PGK) promoter driving either EGFP (FV-SMPGW) or canine PK (cPK) (FV-SM2AGPcPKW). In the FV-SM2AGPcPKW vector EGFP is expressed from the SFFV promoter using a foot and mouth disease virus 2A sequence. **b)** In vitro selection in HT1080 fibrosarcoma cells. After 6 consecutive selections using 50 µM O^6^-benzylguanine (O6BG) and 50 µM *bis*-chloroethyl-nitrosourea (BCNU) the percentage of EGFP-expressing cells is over 80%. The time intervals between treatments was 4, 4, 4, 10, and 6 days respectively. **c)** The tri-cistronic FV vector efficiently transduces canine CD34^+^ colony forming units CFUs (left panel) with a similar efficiency and with similar copy numbers as determined by real-time PCR (right panel) to the control FV-SMPGW vector.
(TIF)
