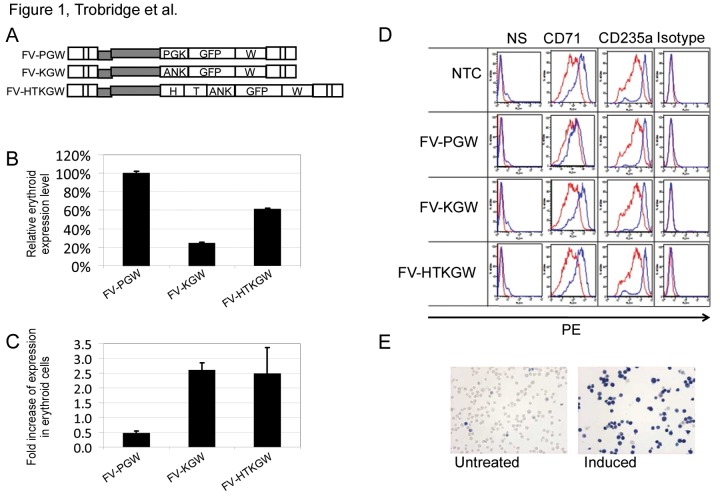# Correction: Stem Cell Selection In Vivo Using Foamy Vectors Cures Canine Pyruvate Kinase Deficiency

**DOI:** 10.1371/annotation/90f278b2-f474-42ec-8645-435f7006018c

**Published:** 2013-10-30

**Authors:** Grant D. Trobridge, Brian C. Beard, Robert A. Wu, Christina Ironside, Punam Malik, Hans-Peter Kiem

Panels D and E are missing from Figure 1. Please see the corrected Figure 1 here: 

**Figure:**