# Supplementary figures and images for: Stem Cell Selection In Vivo Using Foamy Vectors Cures Canine Pyruvate Kinase Deficiency
Source: PLoS One. 2012 Sep 13;7(9):e45173. doi: 10.1371/journal.pone.0045173 (PMC3441638; doi:10.1371/journal.pone.0045173)

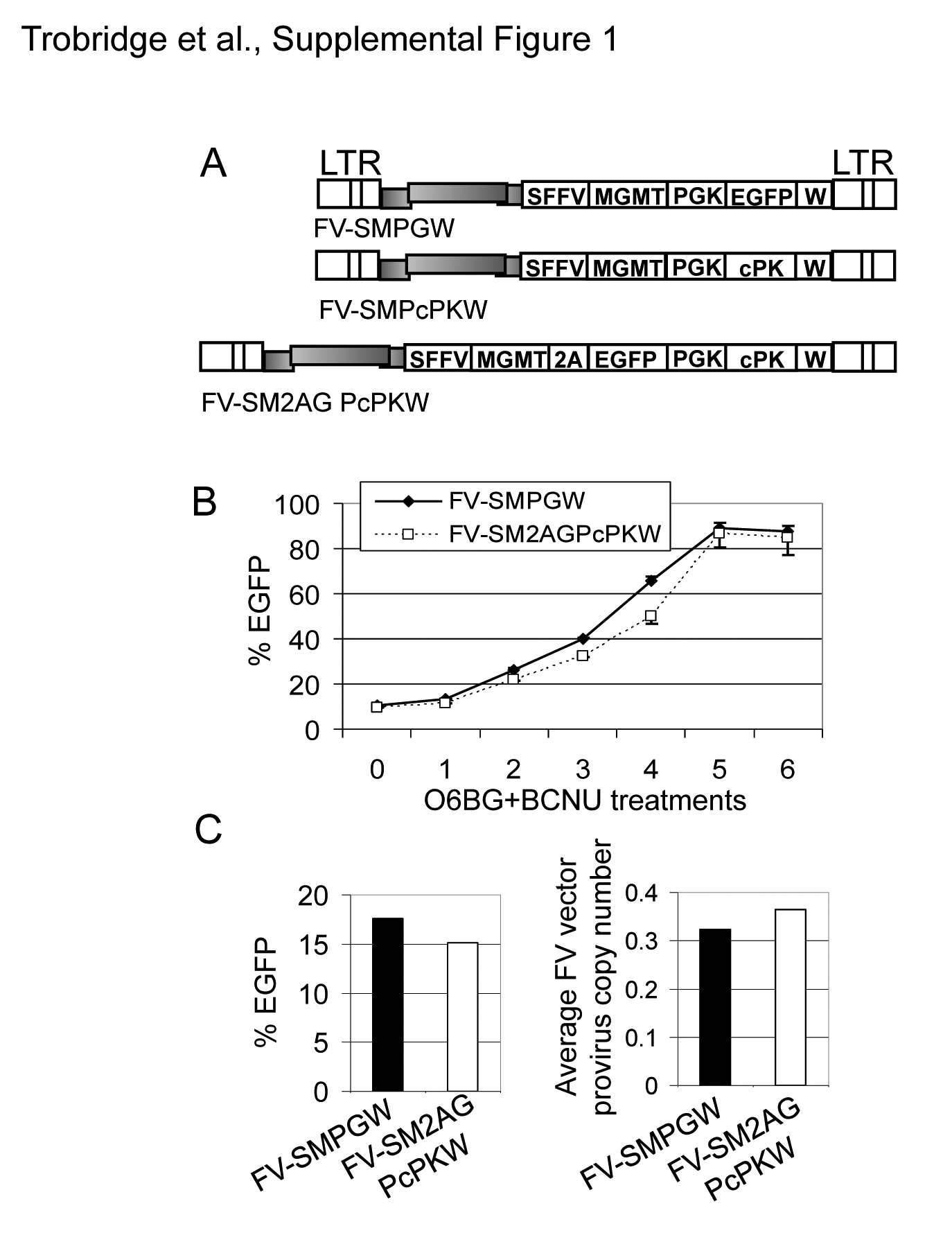

Supplement: Figure S1 — MGMT-mediated selection in vitro with a tri-cistronic FV vector. a) The FV-SMPGW and tri-cistronic FV-SM2AGPcPKW vectors contain the spleen focus-forming virus (SFFV) promoter driving MGMTP140K expression and the phosphoglycerate kinase (PGK) promoter driving either EGFP (FV-SMPGW) or canine PK (cPK) (FV-SM2AGPcPKW). In the FV-SM2AGPcPKW vector EGFP is expressed from the SFFV promoter using a foot and mouth disease virus 2A sequence. b) In vitro selection in HT1080 fibrosarcoma cells. After 6 consecutive selections using 50 µM O6-benzylguanine (O6BG) and 50 µM bis-chloroethyl-nitrosourea (BCNU) the percentage of EGFP-expressing cells is over 80%. The time intervals between treatments was 4, 4, 4, 10, and 6 days respectively. c) The tri-cistronic FV vector efficiently transduces canine CD34+ colony forming units CFUs (left panel) with a similar efficiency and with similar copy numbers as determined by real-time PCR (right panel) to the control FV-SMPGW vector. (TIF) [file pone.0045173.s001.tif]
